# scSE-NL V-Net: A Brain Tumor Automatic Segmentation Method Based on Spatial and Channel “Squeeze-and-Excitation” Network With Non-local Block

**DOI:** 10.3389/fnins.2022.916818

**Published:** 2022-05-27

**Authors:** Juhua Zhou, Jianming Ye, Yu Liang, Jialu Zhao, Yan Wu, Siyuan Luo, Xiaobo Lai, Jianqing Wang

**Affiliations:** ^1^School of Medical Technology and Information Engineering, Zhejiang Chinese Medical University, Hangzhou, China; ^2^The First Affiliated Hospital, Gannan Medical University, Ganzhou, China

**Keywords:** automatic segmentation, non-local block, attention mechanism, deep learning, brain tumor

## Abstract

Intracranial tumors are commonly known as brain tumors, which can be life-threatening in severe cases. Magnetic resonance imaging (MRI) is widely used in diagnosing brain tumors because of its harmless to the human body and high image resolution. Due to the heterogeneity of brain tumor height, MRI imaging is exceptionally irregular. How to accurately and quickly segment brain tumor MRI images is still one of the hottest topics in the medical image analysis community. However, according to the brain tumor segmentation algorithms, we could find now, most segmentation algorithms still stay in two-dimensional (2D) image segmentation, which could not obtain the spatial dependence between features effectively. In this study, we propose a brain tumor automatic segmentation method called scSE-NL V-Net. We try to use three-dimensional (3D) data as the model input and process the data by 3D convolution to get some relevance between dimensions. Meanwhile, we adopt non-local block as the self-attention block, which can reduce inherent image noise interference and make up for the lack of spatial dependence due to convolution. To improve the accuracy of convolutional neural network (CNN) image recognition, we add the “Spatial and Channel Squeeze-and-Excitation” Network (scSE-Net) to V-Net. The dataset used in this paper is from the brain tumor segmentation challenge 2020 database. In the test of the official BraTS2020 verification set, the Dice similarity coefficient is 0.65, 0.82, and 0.76 for the enhanced tumor (ET), whole tumor (WT), and tumor core (TC), respectively. Thereby, our model can make an auxiliary effect on the diagnosis of brain tumors established.

## Introduction

People are used to calling intracranial tumors as brain tumors. It includes primary brain tumors, that occur from the brain parenchyma, and secondary brain tumors, that metastasize from other parts of the body to the brain. However, because the brain tumor location is inside the brain, it can easily cause nervous system dysfunction. Therefore, no matter how benign or malignant the brain tumor is, it is exceedingly invasive to the human body. Once the brain tumor is found, the usual medical treatment is resection. For example, Gamma Knife is used to treat different brain lesions, often inaccessible for conventional surgery (Militello et al., 2015). However, because of the distinctiveness of brain tumor location, the brain tumor treatment scheme is asked to protect the surrounding tissues to a great extent during the treatment. As you can see, the risk of brain tumor resection is very high, and it can be hard to cut off the tumor completely. Thus, brain tumor has become one of the most lethal cancers (He et al., 2016; Gragert et al., 2018). According to the relevant statistical data in “the Lancet Neurology” in 2019, we can know that there were 329,673 central nervous system cancer cases worldwide in 2016; among these data, there were 106,207 cases and 59,120 deaths in China (Gbd 2016 Brain and Other Cns Cancer Collaborators, 2019). Besides, these cases are increasing year by year. How to treat brain tumors effectively and timely is a hot topic in the medical field of China and even the world.

In diagnosing and treating brain diseases, nuclear magnetic resonance imaging (MRI) technology is one of the most popular medical imaging technologies, with spatial coding and reconstruction technology as its core (Zhao et al., 2017). It is widely used because its technology is harmless to the human body, and it has the characteristics of high image resolution. However, although MRI image has many advantages in the auxiliary diagnosis of diseases, the manual segmentation of brain tumors based on MRI image consumes many human and material resources and has the situation of misdiagnosis and missed diagnosis due to the influence of the location of the brain tumor, shape, texture or other features of the brain tumor, and heavy dependence on experts’ professional knowledge and experience in artificial segmentation method (Menze B. et al., 2015). Though MRI image has many advantages in the auxiliary diagnosis of diseases, the manual segmentation of brain tumors based on MRI image consumes many human and material resources and has the situation of misdiagnosis and missed diagnosis (Menze B. H. et al., 2015). Thus, it can be seen that using computer vision based on deep learning to improve the analysis accuracy and processing efficiency of brain tumor MRI image segmentation plays an essential role in the timely and effective treatment of brain tumor patients.

**FIGURE 1:**
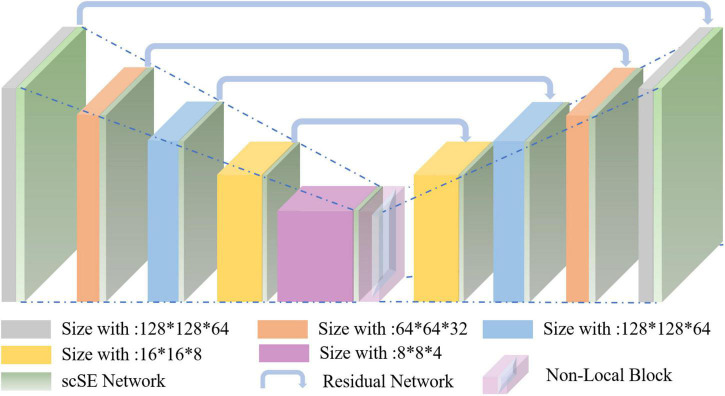
Schematic representation of the proposed scSE-NL V-Net architecture.

Nowadays, the deep learning algorithm is increasingly popular in medical image analysis and is gradually applied to brain tumor segmentation, such as the fully convolutional networks (FCN) proposed by Long et al. (2015). However, most of the current segmentation algorithms are for two-dimensional (2D) image segmentation, which could not effectively obtain the spatial dependence between features. We put forward a method based on spatial and channel “Squeeze-and-Excitation” network with the non-local block to solve the above problem. The scSE-NL V-Net structure used in this paper is shown in Figure 1.

The improvements of our model include the following: (1) Efficiently improving the network’s ability to obtain remote feature dependence. (2) Strengthen the convolutional neural network (CNN) network’s image recognition ability. (3) Achieve a higher segmentation effect.

## Related Works

### Traditional Machine Learning-Based Methods

In the case that deep learning algorithms are not very popular, traditional machine learning algorithms are widely used in medical image segmentation. Some simple threshold segmentation algorithms, such as the Potts model proposed by Doyle et al. (2013), achieved the optimal segmentation results by constraining the current pixel category according to the domain pixel category. Jafarpour et al. (2012) used the gray matrix co-occurrence to describe the edge features of MRI and used PCA + LDA for feature selection to automatically classify normal brain and abnormal brain MRI images. Sijbers et al. (1997) presented a three-dimensional (3D) variant of the watershed algorithm based on Vincent and Solis immersion, which applied the 3D adaptive anisotropic diffusion filter to MR data in advance and improved the shortcomings of the watershed algorithm about over-segmentation. Furthermore, Boykov et al. (2002) reduced over-segmentation by incorporating small volume primitives with similar gray-level distributions. Militello et al. (2015) proposed an unsupervised semi-automatic segmentation method based on the fuzzy C-means clustering, which can assist in segmenting the target and automatically calculate the lesion volume. Huang et al. (2017) put forward an SDAE model to stratify the clinical risks of ACS patients. Others, such as Leibardo et al. (2018), used the GTVcut semi-automatic seed image segmentation method based on the cellular automata model; using graph-based segmentation to find local minima efficiently, and through these two moving methods, the label value of the pixel can be adjusted arbitrarily, so that implement classification. Chen et al. (2019) proposed a novel hybrid model for ITE estimation bridging multi-task deep learning and *K*-nearest neighbors (KNN). Zeng et al. (2020) put forward a dynamic-neighborhood-based switching particle swarm optimization (PSO) algorithm that hybridized the differential evolution algorithm with the PSO algorithm to alleviate premature convergence. For the PSO algorithm, Luo et al. (2020) also proposed a new segmentation method based on graphs, widely used in image restoration, stereo, and motion. An expectation–maximization (EM) algorithm is used to model the nano-gold immunochromatographic assay, from which, the model parameters, the actual signal intensities of the test and control lines as well as the noise intensity are identified simultaneously (Zeng et al., 2013).

### Deep Learning-Based Methods

With computer hardware innovation, the computing power and data capacity have been improved, and various new algorithms have been proposed. In artificial intelligence, multiple algorithms represented by deep learning have made breakthrough in computer vision one by one. The development of deep neural networks (DNNs) gives birth to a series of excellent research results in the same period (Long et al., 2017; Badrinarayanan et al., 2019). Havaei et al. (2017) used 2D CNNs as the segmentation method for brain tumor MRI images. They consciously enhanced the global feature extraction of their model. Moreover, the convolution implementation of the full connection layer is used to improve efficiency. However, they did not pay more attention to the feature association information on the dimension. Long et al. (2015) improved CNN by replacing full connection layers with convolution layers and proposed an end-to-end image semantic segmentation method named fully convolutional networks (FCN). They were trying to improve the model’s segmentation performance by combining semantic and appearance information. Hamghalam et al. (2020) used the general lateral network (GAN) as a brain tumor segmentation model. Through the GAN, they extended to synthesize images with high contrast. As a result, the actual channel number of MR segmentation can be reduced. Furthermore, Zhang et al. (2019) proposed the attention-guided network (AG-Net) model by using filters to detect sensitive structures. An attention module is also added to the model to eliminate noise interference. The experimental results proved that adding a self-attention block could improve segmentation accuracy to a certain extent. Zeng et al. (2021) put forward a novel deep belief network (DBN)-based multi-task learning algorithm to help classify Alzheimer’s disease (AD) and mild cognitive impairment (MCI). Regarding feature information extraction and utilization, Zeng et al. (2022) proposed a method called “the atrous spatial pyramid pooling-balanced-feature pyramid network” (ABFPN). This enhanced multi-scale feature fusion method uses the context features by atrous convolution operators with different expansion rates and is achieved with skip connection. It can help to improve the target detection performance.

### Our Study on Brain Tumor Segmentation

Fully convolutional neural networks (FCN) have become one of the most popular image segmentation tools in the field of medical imaging (Ronneberger et al., 2015; Roy et al., 2017, 2018a) and computer vision (Noh et al., 2015). Convolution network works extract image features with ergodic sliding of convolution on the feature map. Therefore, although the convolution network can effectively extract the feature information of the image, the extracted feature information’s receptive field will be limited by the size of the convolution kernel, and only local feature information can be obtained. Generally speaking, the size of the 2D convolution kernel used in the 2D model is 3 × 3 or 5 × 5, so these CNN-based network structures are complicated to capture feature dependence in a long range. Similarly, the problem of the remote feature dependence capture is still not well solved in the 3D-CNN model.

In this paper, we propose to add a “Spatial and Channel Squeeze-and-Excitation” Network (scSE-Net) to the V-Net model to calibrate CNN image feature sampling area, expected to improve CNN image recognition. We optimize the performance of the V-Net model in obtaining remote feature information by adding a non-local block. We adopt volume input instead of slice input in terms of data input and use 3D convolution to process MRI images.

The innovations of this paper’s model are as follows:

• The use of a self-attention mechanism to improve the network’s ability to obtain remote feature dependence.
• Spatial and channel “Squeeze and Excitation” used to strengthen the CNN network’s image recognition ability.
• Using multi-modal brain tumor segmentation challenge (BraTS) 2020 dataset and achieve a higher segmentation effect.

## Materials and Methods

**FIGURE 2:**
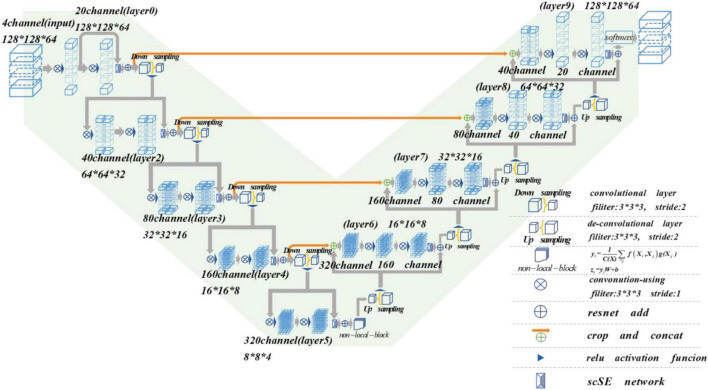
Schematic representation of proposed scSE-NL V-Net architecture.

The MRI image segmentation model used in this paper is 3D V-Net and is based on scSE-network and combined with non-local block. In this section, we will introduce the 3D V-Net, scSE-network, and non-local block. The architecture of scSE-NL V-Net is shown in Figure 2.

### V-Net Framework

With the continuous development of deep learning computer vision technology, the CNN network is more and more widely used in the field of medical image analysis. As we all know, CNN performs well in 2D image processing, but most of the medical data in clinical use are 3D. Therefore, this paper uses V-Net as the segmentation model for brain tumor MRI images.

V-Net is a 3D version of U-Net, which is based on fully convolutional neural networks (FCN). It is a 3D image segmentation model using end-to-end training mode. As an improved 3D network architecture of FCN, V-Net also uses a convolution layer instead of a full connection layer to achieve end-to-end image semantic segmentation of 3D medical images.

According to Figure 2, the network is composed of left and right parts. The network’s left side is the encoding path, which extracts the image features we need from MRI images by convolution. We add scSE-network to improve the ability of image recognition and reduce the resolution through a specific step size at the end of each layer. In the last layer of the coding path, we add the non-local block to obtain remote feature information. scSE-network and the non-local block will be described in detail later. The network’s right side is the decoding path, which is used to restore the feature map to its original size. At the end of the network, we use softmax to classify the images to get the segmentation results of the enhanced tumor (ET), whole tumor (WT), tumor core (TC), and background.

Depending on the resolution size of the operation, we divide the network encoding path into five stages. Each stage consists of 1–2 convolution layers with 3 × 3 × 3 convolution kernels. Draw lessons from the residual network (He et al., 2016), at the end of each stage, we add the original feature map to the feature map, activated by convolution and activation function point by point, to learn the residual. After that, downsampling is carried out. In the research of Fausto et al. (2016), it is shown that such a structure can obtain a faster convergence effect than the model without a residual network.

It is mentioned in Springenberg et al. (2014) that using the pooling layer as the downsampling layer will cause loss of the feature map features, and the recognition accuracy will reduce. Thus, we use the convolution layer with the convolution kernel size of 3 × 3 × 3 and step size of 2 as the downsampling layer instead of the pooling layer (Fausto et al., 2016). Through downsampling, the size of the feature map is reduced and the number of channels is doubled to improve the receptive field of the feature.

The decoding path is the same as the encoding path. We divide the decoding path into four stages to recover the feature map’s size and extract the corresponding features for classification. In each location, we use deconvolution to enlarge the size of the feature map first. Then as in Ronneberger et al. (2015), the feature map in the left network is mapped to the feature map in the right side horizontally of the corresponding size to facilitate better feature extraction.

At the end of the network, we use softmax to calculate four categories with the same size as the original image through 1 × 1 × 1 convolution to the ET, WT, TC, and background in MRI images of brain tumors.

In the scSE-NL V-Net, ReLU functions are used as all activation functions (Tanaka, 2018), because it is easy to derive, and the vanishing gradient problem will not occur.

### Non-local Block

**FIGURE 3:**
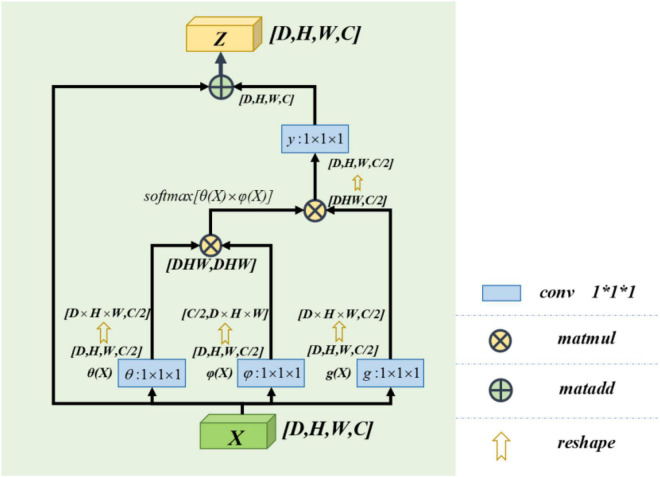
Non-local block structure diagram.

The non-local block adopted in this paper refers to the non-local operation idea put forwarded in “non-local neural networks,” published by Wang et al. (2018). Non-local is a self-attention mechanism. From Wu et al. (2022), the non-local mechanisms can help to extract features effectively. Its core is to capture long-distance feature dependencies through non-local operations to make up for the shortcomings of the small receptive field, low efficiency of long-range feature-dependent capture. A deep network is needed to achieve dependency capture. The non-local block structure is shown in Figure 3.

The calculation equation of the non-local block is as follows:


(1) $$y_{i}=\frac{1}{C\,\left(X\right)}\,\sum_{\forall j}f\,\left(X_{i},X_{j}\right)\,g\,\left(X_{j}\right)$$

(2) $$z_{i}=y_{i}\,W+b$$

where “*X*” is the input signal (in this paper, feature map is used as an input signal) and “*i*” is the index of output position (in space, time, or space–time). Its response value is calculated by “*j*” enumerating all possible situations. The function “*f*” calculates the similarity relationship between “*i*” and all “*j*,” and the unary function *g*(*x*) calculates the representation of the input signal at position “*j*.” The final response value is obtained by standardizing the response factor *C*(*x*) (Wang et al., 2018).

Learn from Wang et al. (2018), this paper defines *C*(*X*) = ∑∀*jf*(*X*_*i*_,*X*_*j*_). So the computing equation we get for non-local computing is *y*_*i*_ = *softmax*[*f*(*X*_*i*_,*X*_*j*_)]*g*(*X*_*j*_). It satisfies the self-attention relation in the field of computer vision (Vaswani et al., 2017). Again in Vaswani et al. (2017), it can be known that the concrete form of function *g*(*x*) and *f*(*X*_*i*_,*X*_*j*_) has little effect on the final performance of self-attention. In order to simplify the operation, 1 × 1 × 1 convolution kernel is used to make linear processing for *X*. The computing equation is as follows:


(3) g⁢(x)=x⁢W+b

(4) $$f\,\left(X_{i},X_{j}\right)=\theta \,\left(X_{i}\right)\,\varphi \,{\left(X_{j}\right)}^{T}$$

(5) $$\theta \,\left(X\right)=X\,W_{\theta }^{T}+b_{\theta }$$

(6) $$\varphi \,\left(X\right)=X\,W_{\varphi }^{T}+b_{\varphi }$$

In our model, we use a feature map as the input signal *x* of the non-local block. Note the depth, width, height, and number of channels of the feature map as *D*, *W*, *H*, and *C*, respectively. First, we process *X* by setting the number of convolution kernels to half of the input feature map channels. So that we can get the number of channels of *C*/2,θ(*X*),φ(*X*), and *g*(*X*) with constant depth, width, and height. The purpose of this is to reduce the amount of computation without affecting the performance of the non-local block. After that, we multiply the θ(*X*) and φ(*X*) matrices while the shape of θ(*X*) and φ(*X*) is changed as [*dwh*,*c*/2] and [*c*/2,*dwh*] to complete the non-local self-attention mechanism in this way. The result is multiplied by *g*(*X*), which is also transformed into [*DWH*, *C*/2] by the morphological transformation. It is then convoluted by 1 × 1 × 1, whose number of convolution kernels is *C*, and the output with the same size as the original feature map is obtained.

**FIGURE 4:**
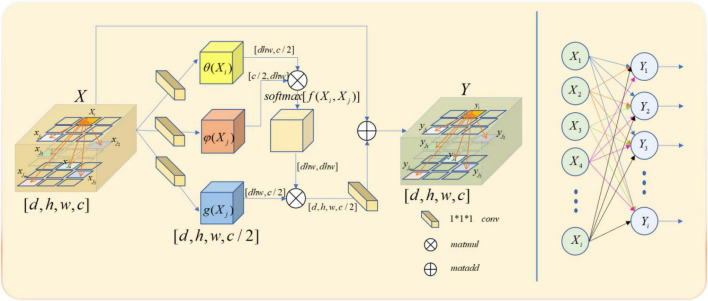
Comparison between non-local block **(left)** and full connection layer **(right)**.

Though both non-local and full connection layers need to compute the entire feature map to get the required features, it is evident that non-local has more advantages than the full connection layer in accurate feature extraction. The full connection layer needs to change its morphology into a list of neurons with fixed numbers, size, and loss of part of location information, when the non-local output is the same size with the original feature map. The comparison between non-local operation and full connection layer operation is shown in Figure 4.

### scSE-Network

**FIGURE 5:**
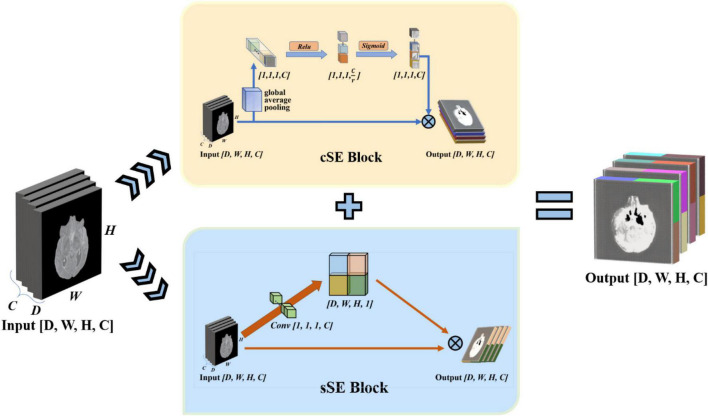
Introduction of spatial and channel “squeeze-and-exception” block. The scSE-block is composed of cSE-block and sSE-block. In scSE-block, the cSE-block and sSE-block have the same input. The output is *cOutput* add to *sOutput*.

To enhance the model’s perception of feature information in both spatial and channel directions, we draw on the experience of the idea of the Squeeze-and-Excitation Networks. This network proposed a mechanism to let the network learn to use the importance of global information to highlight features that may be useful and suppress features that are not so useful (Hu et al., 2017). We use the spatial and channel “Squeeze-and-Excitation” network (scSE-Net) (Roy et al., 2018b) to optimize our model. The frame of the spatial and channel “Squeeze-and-Excitation” network is shown in Figure 5.

scSE-Net is composed of spatial squeeze and channel excitation (cSE) block and channel squeeze and spatial excitation (sSE) block. In scSE-block, the cSE-block and sSE-block have the same input. The output is *cOutput* add to *sOutput*. For the cSE block, it is the original squeeze-and-excitation networks (SE-Nets). And the block sSE are changed from SE-Net. In SE-Net, the most crucial operation is squeeze and excitation (Hu et al., 2017).

#### Squeeze in Squeeze-and-Excitation Network

First, in order to solve the local dependence of features, we use global average pooling (Shen et al., 2015) as the squeeze operation in order to compress the input feature map according to the spatial dimension, and change the size of the feature map from *D*×*W*×*H*×*C* to 1×1×1×*C*. The calculation equation of squeeze operation is as follows:


(7) $$s_{c}=F_{s}\,\left(X_{c}\right)=\frac{1}{D\times W\times H}\,\sum_{i=1}^{D}\sum_{j=1}^{W}\sum_{k=1}^{H}X_{c}\,\left(i,j,k\right)$$

#### Excitation in Squeeze-and-Excitation Network

After squeezing operation, all the network get is the global information on the channel, which cannot explain the channel’s weight well. To get the channel’s level dependency better, we need to do the excitation operation. The excitation operation needs to be flexible enough to learn the non-linear interaction and non-mutex relationship between channels to allow multiple channels to be activated simultaneously (Hu et al., 2017). For this reason, we use two fully connected layers. The first fully connected layer is started by the ReLU function, and the second fully connected layer is activated by the sigmoid function. The calculation equation of excitation operation is as follows:


(8) $$s\,e_{c}=F_{e}\,\left(s_{c},W\right)=R\,e\,l\,u\,\left(g\,\left(s_{c},W\right)\right)=R\,e\,l\,u\,\left(W_{2}\,S\,i\,g\,\left(W_{1}\,s_{c}\right)\right)$$

In the calculation Equation 8, $W_{2}\in {\mathbb{R}}^{C\times \frac{C}{r}},$$W_{1}\in {\mathbb{R}}^{\frac{C}{r}\times C},$ *C* means the number of channels, *r* means scaling parameters, which is mainly used to reduce the computational complexity and parameters of the network. In this paper, the *r* is set to 4.

The final step of SE-Net is to fuse the obtained feature map with the original feature map to let the output with the same size as the original feature map. The cSE block flow chart is shown in Figure 5. The fusion equation is as follows:


(9) $$\hat{X}=F_{S\,E}\,\left(X_{c},s\,e_{c}\right)=s\,e_{c}\times X_{c}$$

#### Channel Squeeze and Spatial Excitation

Through the cognition of SE-Net, we know that SE-Net compresses the space along the channel’s direction and then excites each other with the channel as the unit. The sSE block assume that mutual excitation between spaces is equally important. Therefore, we need to compress the input feature map. Then, the obtained spatial information is activated by the sigmoid function. Finally, we fuse the acquired feature map with the original feature map to complete the sSE block. The sSE block flow chart is shown in Figure 5. The calculation equation of the sSE block is as follows:


(10) $$\tilde{X}=F_{s\,S\,E}\,\left(X_{v},s\,e_{s}\right)=s\,e_{s}\times X_{v}=S\,i\,g\,\left(W_{s},X_{v}\right)\times X_{v}$$

In Equation 10, *se*_*s*_ ∈ ℝ^*D*×*W*×*H*×1^ and *W*_*s*_ ∈ ℝ^1×1×1×*C*^,$\tilde{X}$ means the final output for sSE block.

#### Spatial and Channel “Squeeze-and-Excitation” Block

scSE block is a combination of cSE block and sSE block. The function is $X_{s\,c\,S\,E}=\hat{X}+\tilde{X}.$ The purpose is to obtain the weight of the importance of spatial information and channel information to optimize the model and realize the perception of feature information in both spatial and channel directions.

## Experimental Setup

### Database and Training Environment

To verify the feasibility of the proposed method, we use the BraTS2020 database to test our model (Bakas et al., 2017, 2018). In the BraTS2020 database, all the data are MRI image stored in NIFTI format. There are four sequences available for each patient data in BraTS2020: T1-weighted (T1), T1 with gadolinium-enhancing contrast (T1ce), T2-weighted (T2), and fluid-attenuated inversion recovery (Flair). And correspondingly, each patient data have a mask.nii file to store correct results which were segmented and marked manually.

In the BraTS2020 dataset, all the MRI images were processed co-registered, skull-stripped, and interpolated. All the dimensions of the MRI images have 240 × 240 × 155 voxels (Zhou et al., 2020), and the size of spacing is 1 × 1 × 1. The same as the training data, the dimensions of the mask data we get are 240 × 240 × 155 voxels. Mask data have four labels totally: “0” implies background, “1” implies TC area, “2” implies ET area, and “4” means WT area. The challenge’s official data are divided into two subsets: a training set and a verification set. In the training set, we have 369 samples, and in the verification set, we have 125 samples. However, we do not have official mask data for the validation set. Consequently, we could not use those sets to test our model. In order to adjust and verify our model better, we randomly selected 15 samples from the 369 samples in the training set as the test set to test our model. The samples which are in the test set do not participate in model training but are used to test the trained model.

The experimental environment is based on TensorFlow 1.13.1, Python 3.6.5, and PyCharm, with the processor of Inter(R) Xeon(R) Silver 4210 CPU at 2.20 GHz, operating system of 64-bit Windows 10, 32 GB RAM, and graphics card of ASPEED Graphics Family (WDDM) and NVIDIA TITAN RTX.

### Data Preprocessing

As the preparation work before model training, the quality of data preprocessing will significantly affect the final accuracy of the model. Influenced by the inhomogeneity of intensity, the change of range and contrast, as well as the noise, the automatic segmentation of MRI brain tumor datasets is a great challenge. Thereby, before inputting the dataset into the model, we need to preprocess the dataset. Generally speaking (Akkus et al., 2017), the preprocessing for the MRI image dataset includes image registration, skull stripping, bias field correction, intensity normalization, and noise reduction.

This paper has already introduced that each data in the training set have four MRI successions of different modalities and a mask file used to store manual mark segmentation results. Since the BraTS2020 dataset provided by the official challenge has already been processed by image registration and skull stripping, we just need to do the remaining operations. After researching, we decided to use the *Z*-score method to standardize each model image for MRI dataset’s preprocessing. The *Z*-score method can make the gray value of the image conform to the standard normal distribution and change the standard deviation to 1, so as to improve the data comparability and weaken the noise influence on the image features. The calculation equation of the *Z*-score is as follows:


(11) $$z\,s=\frac{\text{x}-\mu }{\sigma }=\frac{\text{x}-\mu }{\sqrt{\frac{1}{N}\,\sum_{i=1}^{N}{\left({\text{x}}_{i}-\mu \right)}^{2}}}$$

In Equation 11, “zs” means *Z*-score values, “μ” means the mean of image gray value, and “σ” means the standard deviation of image gray value.

**FIGURE 6:**
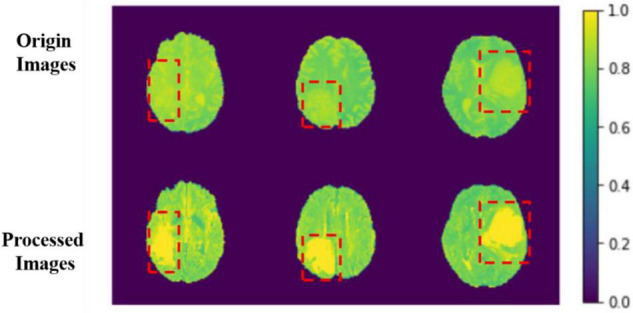
Comparison between origin images and processed images. The red boxes are the approximate area of brain tumor framed after comparing with the mask.

The comparison results between the preprocessing and the original image are shown in Figure 6.

The image features become more evident after processing, and the features of images can be learned for the model more easily. We sample each MRI image and process them into several MRI images with the width, height, and depth of 128, 128, and 64. With this, we can use those data as model input more efficiently.

First, as the MRI images were too big and difficult for our machine to learn the feature, we need to segment the MRI images to proper size. By calculation, we decided to make a patch of every 25 pixel width, 25 pixel height, and 15 pixel depth on MRI images. The method aims to reduce the number of samples and make sure to keep the integrity of features in MRI images. Moreover, more data would be acquired for better model training.

**FIGURE 7:**
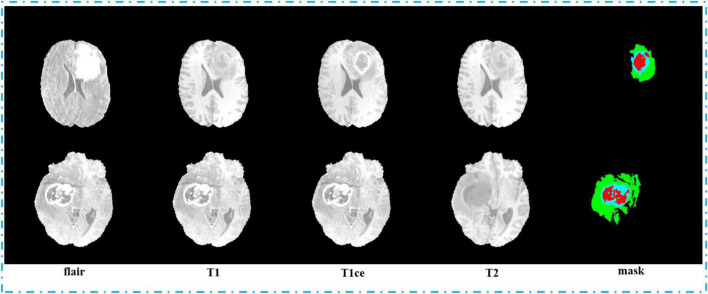
View of four sequences (flair, T1, T1ce, and T2) and the mask.

Second, we pack the MRI images we got by sampling. We pack the MRI images with flair, T1, T1ce, and T2 sequences into four channels and 3D images with the shape of 64, 128, 128, and 4, and they are saved in npy format. In order to label the mapping relationship of data in training, we give each of our sample a unique sequence number. We guarantee that each sample image corresponds to a mask image with the same sampling sequence number whose shape is 64, 128, and 128. We save mask images in npy format too. The view of the four sequences and mask is shown in Figure 7. In order to make the area occupied by each part of the mask more clearly, we restore the mask and predict result to the brain tumor image and represent the WT, the ET, and the TC with green, blue, and red colors, respectively.

### Loss Function

In this paper, we choose the Dice coefficient as the loss function of our model. The Dice coefficient is a function to calculate the similarity of sets, and its range is 0, 1. Its definition equation is as follows:


(12) $${}_{D\,i\,c\,e=\frac{2\times \left|Y_{p\,r\,e\,d}\,⋂Y_{g\,t}\right|}{\left|Y_{p\,r\,e\,d}\right|+\left|Y_{g\,t}\right|}}$$

According to the equation definition, we can get the prediction effect of the model for MRI image segmentation. The equation is gained by the ratio of the area of the coincidence area between the model’s predicted value and the real value of the data and the sum of the area of the predicted value and the data’s actual value. The closer the Dice coefficient is to 0, the worse the segmentation effect is; the closer to 1, the better the segmentation effect is.

### Training Process

In Wang et al. (2018), we learned that the location of the non-local block has little effect on the final segmentation or classification result of the network. Considering the computing power of the computer and the amount of computation of the model, we decide to place the non-local block on the last layer of the model coding path. To better explain the architecture of scSE-NL V-Net, which we used in this paper, we list relevant parameters of the network, as shown in Table 1.

**TABLE 1:** The parameters in scSE-NL V-Net.

Model layers	Type	Filter size/number/layers	scSE block	Non-local block	Active function	Input (D × W × H × C)
Layers 0	Conv	3 × 3 × 3/20/1	No	No	ReLU	64 × 128 × 128 × 4
Layers 1	Conv	3 × 3 × 3/20/1	Yes	No	ReLU	64 × 128 × 128 × 20
Layers 2	Conv	3 × 3 × 3/40/2	Yes	No	ReLU	32 × 64 × 64 × 40
Layers 3	Conv	3 × 3 × 3/80/2	Yes	No	ReLU	16 × 32 × 32 × 80
Layers 4	Conv	3 × 3 × 3/160/2	Yes	No	ReLU	8 × 16 × 16 × 160
Layers 5	Conv	3 × 3 × 3/320/2	Yes	Yes	ReLU	4 × 8 × 8 × 320
Layers 6	Conv	3 × 3 × 3/160/2	Yes	No	ReLU	8 × 16 × 16 × 160
Layers 7	Conv	3 × 3 × 3/80/2	Yes	No	ReLU	16 × 32 × 32 × 80
Layers 8	Conv	3 × 3 × 3/40/2	Yes	No	ReLU	32 × 64 × 64 × 40
Layers 9	Conv	3 × 3 × 3/4/2	Yes	No	ReLU	64 × 128 × 128 × 20

## Experimental Results

After 420,000 steps of training, we use the trained model to test the actual prediction effect. As mentioned above, since the official verification set does not mask target information, we randomly selected 15 samples in the training set, which are not used for training, but only for testing and evaluating the performance of the models as well as using for following visualization effect displaying.

**FIGURE 8:**
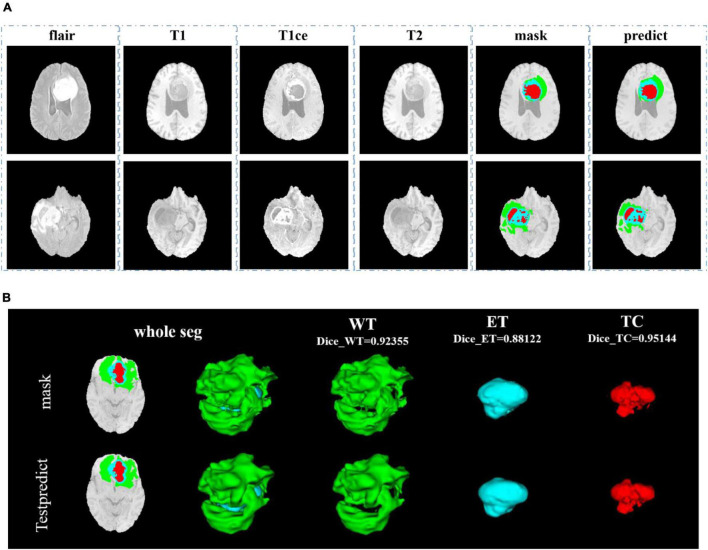
Comparison of visualization results between the original sample, mask, and prediction segmentations is shown in panel **(A)**. Panel **(B)** shows the 3D segmentation effect of our model.

In Figure 8, we show the visualization result of two random samples, including the comparison of visualization results between the original sample, standard manual segmentation, and prediction segmentation. To observe the effects of brain tumor segmentation more clearly and intuitively, we restore the mask and predict result to brain tumor image and represent the WT, the ET, and the TC, with green, blue, and red colors, respectively.

**FIGURE 9:**
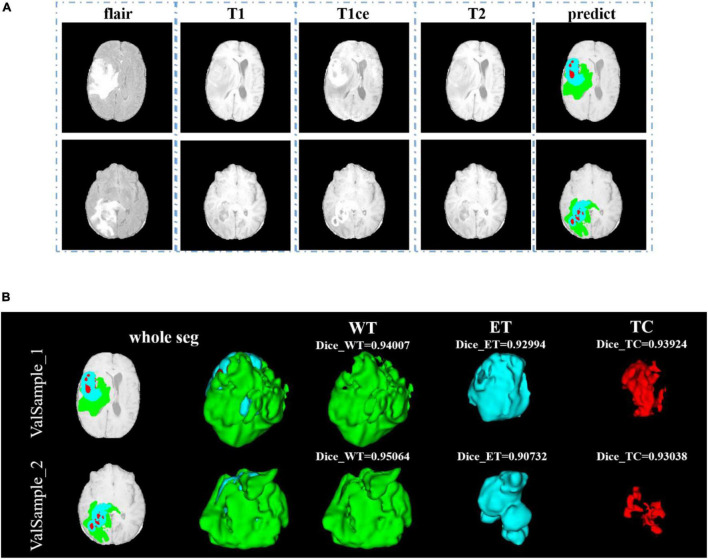
Comparison of visualization results between the original sample, and prediction segmentations is shown in panel **(A)**. Panel **(B)** shows the 3D segmentation effect of our model.

After training, we use the model to segment the tumor region from the official verification set and send the segmentation results to the official platform to verify the model’s effectiveness. In the verification set, we also randomly select two samples to show the visualization results. The visualization result of the validation set is shown in Figure 9. Since the official verification set has no mask tag, we did not show the comparison between the target and the prediction in the visualization results.

In Figure 9, we use the same approach as in Figure 8. From Figures 8, 9, in the random sampling prediction, the results obtained by our model are very similar to the given labels in terms of overall and details. The model can help medical diagnosis to a certain extent and save a lot of human and material resources.

**FIGURE 10:**
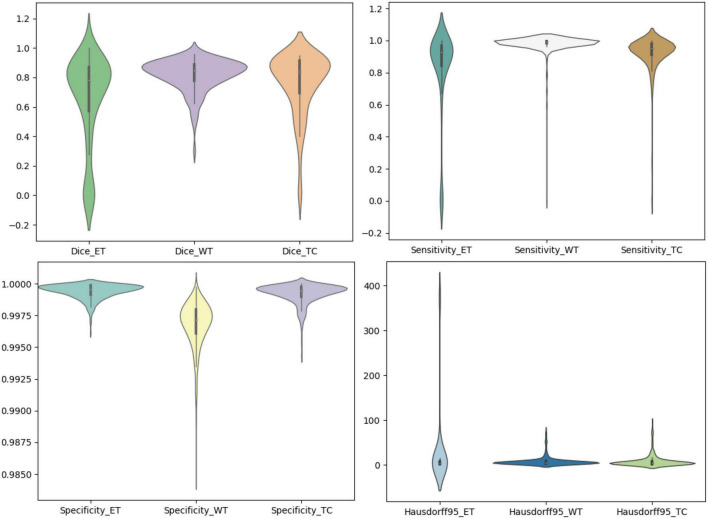
Statistics of performance for brain tumor region segmentation of the training set.

**FIGURE 11:**
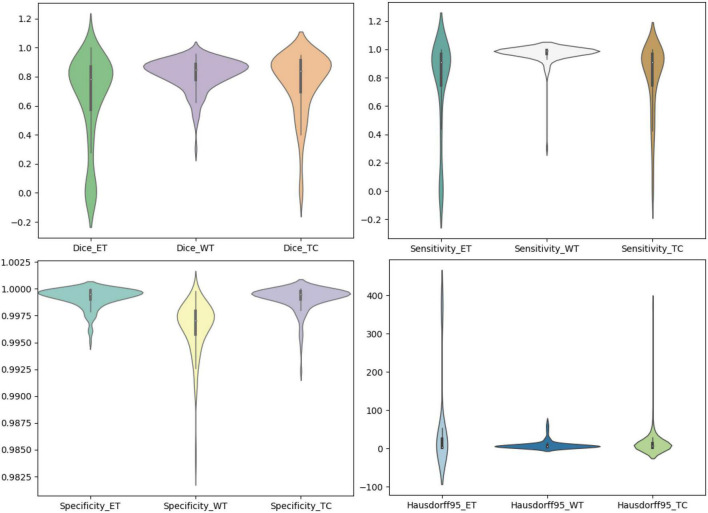
Statistics of performance for brain tumor region segmentation of the validation set.

We use the trained model to segment the data in the brain tumor challenge training dataset and validation dataset and upload the segmented results to the data evaluation path given by the challenge official, so as to obtain the statistical data including four evaluation indicators. In Figures 10, 11, we show the performance of scSE-NL V-Net in the segmentation of tumor regions in the training dataset and the validation dataset with the form of violin statistical chart, respectively. In the graph, we set the evaluating indicators as *X*-axis and set their coefficient values as *Y*-axis. The performance of our model is shown in Figures 10, 11.

There are four official evaluation indexes for brain tumor segmentation. Dice coefficient focuses more on the difference between the predict outcomes and the mask labels. Sensitivity describes the proportion of positive cases correctly identified by the model to all positive cases identified by the model. Specificity describes the proportion of negative cases correctly identified by the model to all negative cases identified by the model. The higher the Dice coefficient, sensitivity, and specificity are, the better the performance is. The Hausdorff95 distance measures the distance between true subsets in the metric space.

Comparatively speaking, we pay more attention to the analysis of the Dice coefficient. It can be seen that our model is most sensitive to the WT from the figure. Its Dice coefficient is stable at around 0.9. However, ET and TC are relatively weak, and even the Dice coefficient is 0. We can get similar conclusion from the numerical analysis of other evaluation indexes.

Through dataset analysis, we find that many samples with a Dice coefficient of 0 do not have ET or TC. There is no doubt that it will reduce the evaluation of Dice coefficient on the whole model.

## Discussion

To verify the effectiveness of our model, we compare it with some classical segmentation algorithms. Havaei et al. (2017), proposed a brain tumor segmentation method that was based on DNNs. Stacking 2D convolutions were used in their model to extract global features. They tested it in BraTS2013 challenge. Pereira et al. (2016) used CNNs to segment brain tumor images. By exploring a small 3 × 3 kernel, the model could work against overfitting. Thus, they could support deeper architectures. By using this method in BraTS2015 challenge, they won second place. Kamnitsas et al. (2016) put forward a 3D-CNN with fully connected CRF to segment brain tumor images. In the method, they use a dual-channel structure to support multi-scale input image processing simultaneously. Zhou et al. (2020) achieved a one-pass multi-task network (OM-Net) with a cross-task guided attention (CGA) module. And on this basis, they could integrate segmentation tasks into a depth model. They took part in the BraTS2018 challenge and win second place. In Table 2, we show the comparison between our method with other classical algorithms.

**TABLE 2:** Comparison between the proposed method and other classical algorithms.

Team name	Dice		
	ET	WT	TC
**Proposed model bold**	**0.65**	**0.82**	**0.76**
Havaei et al. (2017)	0.57	0.79	0.68
Pereira et al. (2016)	0.75	0.78	0.65
Kamnitsas et al. (2016)	0.63	0.84	0.67
Zhou et al. (2020)	0.65	0.87	0.64

*Bold terms and values represent the best data in the tables.*

Finally, we compare the Dice coefficient of brain tumor segmentation with the results of some competitors on the official website of the BraTS2020 challenge. The comparison parameters we selected are the mean Dice coefficients of the ET, WT, and TC. Table 3 shows the comparison of the prediction results of the verification set:

**TABLE 3:** BraTS2020 validation dataset accuracy comparison table.

	Dice			Sensitivity			Specificity			Hausdorff95		
Team name	ET	WT	TC	ET	WT	TC	ET	WT	TC	ET	WT	TC
**Proposed**	0.647	0.818	0.759	**0.795**	**0.971**	0.795	**0.999**	0.998	**0.999**	44.4	10.0	14.6
Unet3d-test-sz	0.704	0.836	0.725	0.712	0.867	0.785	0.999	**0.999**	0.999	42.1	10.5	12.3
Ovgu_seg	0.602	0.794	0.681	0.664	0.785	0.674	0.999	0.999	0.999	54.1	12.1	19.1
Mpstanford	0.491	0.717	0.622	0.493	0.813	0.688	0.997	0.988	0.994	61.9	26.0	28.0
AI-Strollers	0.578	0.737	0.615	0.523	0.770	0.623	0.999	0.997	0.998	47.2	24.0	31.5
Uran	0.400	0.779	0.580	0.379	0.755	0.574	0.999	0.999	0.999	51.6	12.2	20.2
LMB	**0.716**	0.825	0.765	0.695	0.766	0.722	0.999	0.999	0.999	37.4	12.3	13.1
Agussa	0.684	**0.889**	**0.776**	0.704	0.907	**0.818**	0.999	0.999	0.999	**36.4**	**8.1**	**12.9**
IIITV	0.210	0.383	0.275	0.263	0.469	0.353	0.997	0.991	0.995	99.0	60.1	66.2
NUUEE410Lab	0.572	0.815	0.707	0.574	0.811	0.710	0.999	0.999	0.999	49.9	17.2	22.9
Iris	0.678	0.863	0.733	0.672	0.902	0.704	0.999	0.999	0.999	44.1	23.9	20.0
Alone	0.608	0.809	0.644	0.639	0.767	0.595	0.999	0.999	0.999	49.1	13.3	18.6

*Bold terms and values represent the best data in the table.*

Since we do not know the model selected by other competitors, we can only compare it with the data information of each team’s submitted result given by the official BraTS2020 competition. It has to be said that this is a pity. From data analysis of the table, the performance of our model is better than those of other competitors’ models in some cases. It shows that our method is effective in improving the accuracy of brain tumor segmentation. As we can see, the study of necrotic substances in the WT has important clinical value in treatment planning and cancer progression evaluation. In Rundo et al. (2018), we know that WT contains pathological necrotic areas, which are usually characterized by hypoxia. There is no doubt that special attention must be paid to these hypoxic areas. These pathological necrotic areas are elated to several aspects of tumor development and growth, which may lead to tumor recurrence and resistance to therapeutic damage (Rundo et al., 2018). So, our method can help segment the tumor area effectively.

## Conclusion

In summary, we put forward a method of brain tumor segmentation based on 3D V-Net. In our network, scSE block is used to excitation our model from channel and space two aspects to calibrate the image feature sampling area of CNN to improve the CNN image’s recognition ability. Simultaneously, as a self-attention module, the non-local block is used to obtain the long-distance feature dependency of the image to optimize the performance of the model. The non-local block not only accelerates the convergence speed of the model but also effectively improves the segmentation accuracy of the model.

By comparing the segmentation results of our model on the official verification set with the quantitative test results on the platform given by the official BraTS2020 challenge, from the visual outcomes and quantitative Dice indicators, our network has achieved better results. The mean of the Dice (ET), Dice (WT), Dice (CT) is 0.647, 0.818, and 0.759, respectively. In contrast, our model has relatively higher accuracy on the WT. However, there are some deficiencies in the ET segmentation.

This paper proves the feasibility of the channel and space squeeze-and-excitation mechanism and the self-attention mechanism of the non-local block in model optimization. It can be further verified in the visualization results of our model. From the visualization of segmentation results, we can see that the quantitative data are not enough to evaluate the model’s segmentation effect completely, but we can get enlighten from it and try to optimize it.

Brain tumor segmentation in the medical field has been a long-term research problem. Therefore, we need to continue to encourage ourselves to do more in-depth research on brain tumor segmentation and contribute to the medical community.

## Data Availability Statement

Publicly available datasets were analyzed in this study. This data can be found here: https://www.med.upenn.edu/cbica/brats2020/data.html.

## Ethics Statement

This study was reviewed and approved by the Institutional Review Board (IRB) of Zhejiang Chinse Medical University. Written informed consent was obtained from the individual(s) for the publication of any potentially identifiable images or data included in this article.

## Author Contributions

JuZ, JY, and XL conceived and designed the study. JuZ, JY, YL, JiZ, and JW contributed to the literature search. JuZ, JY, YW, and XL contributed to data analysis and data curation. JuZ, JY, JiZ, SL, and JW contributed to data visualization. JuZ and YL contributed to software implementation. JuZ, JY, YW, JiZ, SL, and XL contributed to the tables and figures. JuZ, YL, JiZ, YW, XL, and JW contributed to the writing of the report. JuZ, JY, YL, XL, and JW contributed to review and editing. All authors have read and approved the publication of this work.

## Conflict of Interest

The authors declare that the research was conducted in the absence of any commercial or financial relationships that could be construed as a potential conflict of interest.

## Publisher’s Note

All claims expressed in this article are solely those of the authors and do not necessarily represent those of their affiliated organizations, or those of the publisher, the editors and the reviewers. Any product that may be evaluated in this article, or claim that may be made by its manufacturer, is not guaranteed or endorsed by the publisher.
